# Outdoor Natural Versus Built Experiments: A Scoping Review and Methodological Recommendations for Psychological Science

**DOI:** 10.3390/ijerph22111708

**Published:** 2025-11-12

**Authors:** Shahar Almog, Maribel Rodriguez Perez, Meredith S. Berry

**Affiliations:** 1 Department of Health Education and Behavior, University of Florida, Gainesville, FL 32611, USA; mrodriguezperez@ufl.edu; 2 Department of Psychology, University of Florida, Gainesville, FL 32611, USA

**Keywords:** nature, urban, environmental exposure, outdoor experiments, psychological outcomes

## Abstract

The beneficial effects of exposure to nature on health and well-being, including enhanced relaxation and improved mood and attention, are well-established. Less evidence exists on understudied outcomes related to clinical populations (e.g., substance use outcomes, decision-making), mainly from laboratory experiments warranting outdoor studies. The purpose of this scoping review was to review and summarize the rich experimental literature of nature exposure on psychological outcomes, and form updated methodological recommendations for future outdoor basic experiments isolating the effect of nature exposure. Four databases and ten systematic reviews were searched. From 6394 references, 60 articles (reporting experiments or secondary analyses) comparing natural versus control-built environments, utilizing short exposure in the environment, and examining psychological outcomes were included and synthesized. We discuss limitations and innovative approaches and provide methodological recommendations. Future research should recruit large and gender-balanced samples, expand to clinical populations, include baseline measurements, assess individual differences, and investigate behavioral and other outcomes that are sparse in the literature. Researchers might consider expanding the dichotomous green–gray environments, pay attention to the sense of safety and participant masking, and assess and report environmental conditions. These recommendations may facilitate investigating unique outcomes that are missing in the literature, which hold implications for nature-prescription and intervention programs.

## 1. Introduction

Research shows that exposure to nature is beneficial for health and well-being. Several reviews and meta-analyses, reviewing both in-lab and outdoor experimental studies utilizing different designs and interactions with nature, show that overall natural environments improve mood [1,2], reduce physiological (and perceived) stress markers [3,4,5], improve health indices and psychiatric condition symptoms [6], and improve attention and cognitive performance [7]. Simultaneously, researchers highlight the scarcity of targeted research among clinical populations, for example, in the context of pain or substance use [8,9,10].

Psychological outcomes relevant to pain and/or substance using populations such as pain catastrophizing, impulsive decision-making, craving for substances, or behavioral economic demand for substances (i.e., the motivation to purchase and consume substances across different prices) are understudied in the context of nature exposure, and when studied, are commonly in laboratory conditions (e.g., [11,12,13]). While in-lab studies have provided some foundation of evidence for the beneficial effects of visual exposure to nature in tightly controlled conditions, outdoor experiments will allow for the development of ecologically valid and intervention-focused experimentation that is immersive (e.g., olfactory, auditory). Such studies could hold direct real-world implications for treatment and nature prescription (i.e., providers prescribing time in nature for therapeutic purposes [14]). Still, whether outdoor experiments may optimize effects in the aforementioned outcomes beyond those observed in laboratory settings is yet to be determined.

While simulated nature may produce improved health and well-being outcomes [15] and may be advantageous in conditions where real nature is not accessible [16], in general, real exposure to nature produces better outcomes than simulated nature (for meta-analyses, see [1,17]). Thus, to evaluate the effect of exposure to real nature on understudied and unique outcomes (e.g., impulsive decision-making, demand for substances) and examine whether the effect of real nature exposure could be larger than that seen in laboratory conditions, the next stage in translation is to follow with outdoor experiments. While real natural environments provide opportunities that have additional benefits for health and well-being such as physical activity and socializing [18], it is necessary to first evaluate the effect of the natural environment in isolation. The literature on basic field experimentation evaluating the effect of exposure to nature versus built environments on affective and cognitive outcomes is rich and has evolved throughout the years. Future research on understudied outcomes could benefit from updated methodological recommendations synthesized from the rich literature that exists.

Thus, to inform future research with understudied psychological outcomes (e.g., decision-making processes) and exposure to real nature while minimizing confounding factors (e.g., physical activity, socializing), the purpose of the present review was to synthesize the methodological aspects of the literature regarding the effects of outdoor natural versus built environments on psychological outcomes including affective and cognitive outcomes. We aimed to form updated methodological recommendations for future outdoor experiments to advance the field by facilitating and calling for more research on understudied or new clinical-related outcomes, to widen the understanding of the therapeutic potential of exposure to nature.

### Objectives

- Review the literature utilizing a scoping review approach, to identify outdoor experiments (and secondary analyses of outdoor experiments) investigating the effect of short exposure to natural environments compared to built environments on psychological variables assessed by self-reported measures or psychological tasks.
- Synthesize the designs, samples, procedures, outcome measures, controlled variables, analyses, potential mechanisms, and general findings.
- Compare methodologies of studies focused on affective versus cognitive outcomes.
- Identify limitations, gaps, and innovative approaches.
- Form methodological recommendations for future research.

## 2. Materials and Methods

### 2.1. Protocol and Registration

A scoping review approach was chosen to cover a broader search and enable the finding of relevant experimental studies, as well as related secondary analysis studies that may contribute to the recommendations that will inform future outdoor experiments. The protocol of the scoping review was not registered but was developed and is available upon request. The scoping review followed the PRISMA-ScR Checklist [19].

### 2.2. Eligibility Criteria, Information Sources, and Search Strategy

The scoping review included a comprehensive search without limitation on date or type of studies to understand the evolution of the field. Only sources available in English were included in this review. Both a digital and a manual search were conducted. Four databases were electronically searched on 22 September 2022 (i.e., Web of Science, PsycInfo, PubMed, and Psychology and Behavioral Sciences Collection). In addition to the electronic search, ten published reviews/meta-analyses were manually searched for eligible studies [1,2,3,5,6,7,20,21,22,23]. The search strategy included terms describing the outdoor environment (e.g., environment, setting, landscape, area, space), nature condition (e.g., nature, park, forest, greenspace), urban condition (e.g., urban, city, traffic, building), interaction with the environment (e.g., walk, view, visit, contact, expose), outcomes (e.g., mood, anxiety, stress, cognitive, attention), and design-related terms (e.g., experiment, control). The search strategy excluded terms of intense exercise (e.g., exercise, fitness, jog), in-lab methods (e.g., image, video), and children or adolescents. Appendix A presents search terms used in Web of Science.

### 2.3. Selection of Sources of Evidence

Studies were included in this scoping review if they encompassed the following: (a) an experimental design, or secondary analysis of experimental studies, (b) outdoor conditions of a natural environment compared to a built setting control condition, (c) assessments of psychological outcomes (self-reported scales or tasks), regardless of other physiological measurements, (d) adult participants—healthy or clinical samples, and (e) a low-effort, short interaction with the environment (up to 60 min) such as a slow walk or viewing. Studies that included children, images or videos of the environments, other interventions (e.g., meditation), longer exposure (e.g., multiple exposure programs), or more intense exercise (e.g., running) were excluded.

### 2.4. Data Charting Process and Data Items

All sources yielded from the search were downloaded to Covidence, an online tool for systematized reviews (https://app.covidence.org/). Following the removal of duplicates, the study selection was conducted in two stages: title and abstract screening and full-text screening. Data were extracted from all included studies for (1) country and year of publication, (2) sample characteristics including number of participants, percentage of female participants, and general description (e.g., clinical, healthy, students), (3) design characteristics including within/between subjects, washout interval, and timing of measurements (i.e., pre-post), masking, (4) exposure characteristics including type of interaction with environment, duration of exposure, description of environment, and other manipulations (e.g., priming fatigue), (5) outcome measures, scales, and tasks, (6) significance of results of beneficial effects of nature compared to built environments and evidence for deleterious effect in the built environment, (7) other measured variables such as environmental data (e.g., temperature, noise) and personal characteristics (e.g., BMI), and (8) examined mechanism (e.g., mediation analysis).

### 2.5. Synthesis of Results

Statistic descriptives of the data were produced using R Statistical Software (v4.1.2) and RStudio (version 2021.09.0, using the rstatix package [24]). No other statistical analyses were conducted, as the purpose of the review was to focus on methodological considerations and identify limitations, gaps, and innovative experimental approaches rather than assess the strength of evidence.

## 3. Results

### 3.1. Selection of Sources

**Figure 1:**
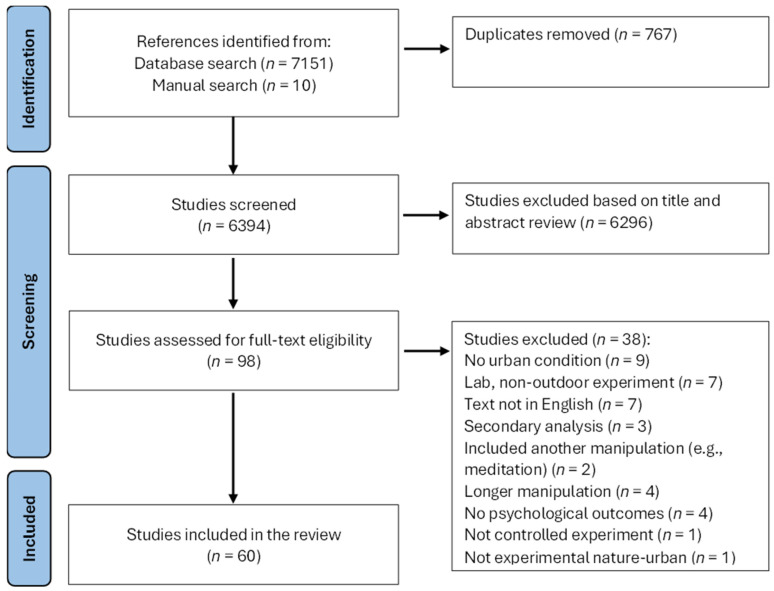
Prisma flow diagram.

The results of the search are presented in the PRISMA diagram in Figure 1. Overall, 7151 references were identified through the digital search and an additional 10 through the manual search. After removing 767 duplicates, 6394 references were screened based on title and abstract, after which 98 were reviewed in full text for eligibility. Finally, 60 articles met inclusion criteria and were included in the review.

### 3.2. Characteristics of Included Studies

The final sample included 60 published articles (herein “studies”) with 62 reported experiments with unique protocols (herein “experiments”, 2 experiments reported in [21,25]. All studies were published in peer-reviewed journals. The earliest study was published in 1991, but most of the studies (75.8%) were published between 2012 and 2022. Of the 60 studies, 32 were conducted in East Asia (mainly Japan with 22 studies), 16 in North America, and 12 in Europe. Table 1 presents the studies’ characteristics.

### 3.3. Synthesis of Findings

The following synthesis summarizes the literature on methodology and results to lay the foundation for forming the recommendations for future research.

#### 3.3.1. Samples Characteristics

Sample sizes were as little as 8 participants [46] and up to 585 participants [75], with a median number of participants of *n* = 32.5 (Q1–Q3: 20–60). Thirty-six (58.1%) experiments included mixed-gender samples. Nineteen experiments (30.6%) included only male participants, and six (9.7%) included only female participants. One (1.6%) did not report the gender of the participants. Most experiments included healthy university students (*n* = 45, 72.6%), while others included adult to older adult community members with or without university students (*n* = 14), or special populations (e.g., adults with depression, hypertension; *n* = 5). The median age of participants overall was 22.6 years (Q1–Q3: 21.2–37.7).

#### 3.3.2. Experimental Procedures

##### Experimental Design

Forty-seven (75.8%) experiments utilized a within-subject design, and fifteen (24.2%) used a between-subject design. The studies that utilized a within-subjects design used a variety of washout periods between sessions. In ten studies (21.3%), the two sessions were conducted on the same day, either without any break or morning/afternoon sessions. In 20 studies (42.5%), the sessions were conducted over two consecutive days, and 17 studies (36.2%) included a longer washout period where the sessions were conducted on non-consecutive days and up to two weeks apart. Of the 62 experiments, 8 (12.9%) specified that the participants were masked from the real purpose of the study, 37 (59.7%) experiments did not mask the participants, and 17 (27.4%) either did not report or were unclear in their report on the level of masking.

##### Interaction with the Environment

Participants were asked either to walk in the environment (*n* = 30), view the environment (*n* = 20, usually in a seated position), or perform some combination of the two (*n* = 12). The duration of the interaction ranged from 5 min and up to 60 min (*Median* = 16, Q1–Q3: 15–30 min). Ten studies (16.7%) employed another manipulation such as priming fatigue (e.g., [30]) or stress (e.g., [55]). Some studies reported on the instructions given to the participants before the exposure. For example, participants were asked to “immerse themselves in the environment” [82] (p. 4), “relax and observe the view” [34] (p. 828), or “relax and be aware of the environment” [42] (p. 18). Additionally, some studies instructed the participants to refrain from using their phones, talking, or socializing with others (e.g., [55,65,67,78]).

##### Environmental Sites and Conditions

Overall, most of the studies dichotomously compared a green environment to a busy urban space. Some studies described the green environment in general terms such as “park” or “forest”; some provided a more specific description, for example, “urban park”, “therapeutic garden”, “coniferous forest”, “urban woodland”, “nature preserve”, or “botanical garden”, sometimes describing the type of vegetation present in the site (e.g., [49]). Examples of the description of the urban environment included “downtown”, “busy street”, “city center”, “street near a railway station”, or “commercial area”. With the goal of investigating whether some nature is more beneficial than others, or the nature dose needed for beneficial effects, several studies focused on specific elements in the environment rather than using “typical” environments. For example, Beil and Hanes [29] included four environments, two green (“mostly natural” and “very natural”) and two urban sites (“mostly built” and “very built”) with different proportions of natural and built elements. Kabisch et al. [48] compared three environments with two natural sites (“old urban park” and “new park”) and an urban control. To utilize more naturalistic conditions, Stigsdotter et al. [78] purposely used a “historic downtown” as control, which may be more restorative than a busy street with traffic and commercial buildings, to better reflect real-world choices of an urban walk for restorative purposes. Similarly, some studies noted that the urban site had vegetation (e.g., [29,40]).

Of the 62 experiments, 66.1% (*n* = 41) assessed and reported some environmental conditions such as weather (e.g., sunny, cloudy), temperature, humidity, wind, noise, illumination, atmospheric pressure, or air pollution. Most of those who reported environmental conditions (*n* = 27) did so for descriptive purposes. Some authors stated that the conditions were comparable (e.g., [43]), while others statistically tested for differences between environments (e.g., [15]). Few assessed environmental conditions as confounders [67,78] or associations between environmental conditions and psychological outcomes (e.g., [48,94]). Twenty-one experiments (33.9%) did not include any information on environmental conditions.

#### 3.3.3. Studies Measures

It is important to note that similar measures were used in many of the studies. This is likely due to a small number of research teams around the world conducting this research and the Shinrin-yoku (or “forest bathing”) studies from Japan paving the way for others.

##### Affective and Restorativeness Measures

All 60 studies assessed affective outcomes, utilizing self-reported measures of mood, stress, restorative effects, and/or others. Examples of most commonly used scales for mood, emotions, and affect were the Profile of Mood States (POMS; [92]) and the Positive and Negative Affect Schedule (PANAS; [91]); for stress and anxiety—the State-Trait Anxiety Inventory (STAI; [101]); and for restorative outcomes—the Subjective Vitality Scale (SVS; [103]), Restorative Outcome Scale (ROS; [94]), or the Perceived Restorative Scale (PRS; [93]). Studies also used the semantic differential method and asked the participants to rate the environment itself on adjective-pair scales (e.g., uncomfortable to comfortable, relaxed to awakening), or qualitative investigation (e.g., [69]). All of these measures assess both positive and negative outcomes, where a positive response to nature is commonly characterized with both an increase in positive outcomes (e.g., positive affect, vigor, vitality) and a decrease in negative outcomes (e.g., negative affect, fatigue, depression, and dejection).

##### Cognitive Measures

Fourteen studies (with 16 experiments) investigated cognitive performance using cognitive tasks. The most commonly used task (administered in seven experiments) assessing directed attention capacity was the Backward Digit Span (BDS). Several other tasks were used, including the Necker Cube Pattern Control Test, Stroop, memory tests, and other tasks assessing cognitive performance (e.g., search task, quiz on reading).

##### Other Measures

Few studies included other outcome measures. Two studies investigated time-related outcomes: time awareness using the Passage of Time Judgement and time estimation tasks [38] and the temporal discounting task (a measure of “impulsive” choice [82]. While in the latter two studies the main interest was the effect of nature on time-related outcomes and the affective outcomes were secondary, few studies included additional secondary measures to complement the affective outcomes. For example, studies examined the effect of nature on rumination (Rumination-Reflection Questionnaire [95] used in [36]), ability to reflect and nature connectedness (Connectedness to Nature Scale [86]), self-awareness (The Situational Self-Awareness Scale [100] used in [54]), creativity [81], and mindfulness (Mindful Attention Awareness Scale [87], used in [27]). Three studies collected qualitative data on the experience in the environment [27,38,69].

Some studies included participant- or environment-related measures that were used to ensure successful randomization and similarity between conditions, or were investigated as factors that could explain the nature effect. For example, participant-related measures included mood disorders, anxiety, substance use [27], area of residence [36], duration of the drive to the experimental location [42], exposure to nature on a daily basis [37], perception of noise [55], absorption and awe [28], nature–urban orientedness and noise sensitivity [56], and baseline stress/trait anxiety [75]. Measures assessing the individual’s relationship with nature included the Environmental Identity Scale ([105] used in [29]), the Inclusion of Nature in Self scale ([106] used in [40]), or the Connectedness to Nature Scale ([86] used in [54]). For clinical populations, specific and relevant factors were collected, for example, for people with depression, factors such as living situation (alone/with others), occupation (yes/no), or depressive symptom severity [83]. Body mass index (BMI) was assessed for people with hypertension [74]. Environment-related measures included measures such as temperature, humidity, sound level, air pollution, etc. (e.g., [32,63,67]).

#### 3.3.4. Studies Analyses

Nineteen experiments (30.6%) assessed the outcome measures post-exposure only, and forty-three (69.4%) assessed the outcomes pre- and post-environmental exposure. Pre-post measurements allow for different types of analysis, including an assessment of the effect of the urban environment. Although some studies assessed outcomes pre- and post-environment exposure, not all evaluated or reported on the effect of the urban environment. Some studies compared the environments on baseline scores (i.e., pre-nature vs. pre-urban) and on the post-exposure scores (i.e., post-nature vs. post-urban) separately (e.g., [53]). Others compared pre versus post scores (e.g., [39]). Some studies used analysis of variance (ANOVA) models to identify differences across environment and time (e.g., [33,36]). The pre-exposure measurement was sometimes used to ensure there were no differences between groups, and later, only the post measurements were used (and reported) for comparison between environments (e.g., [42]). In other studies, if there were differences already existing at baseline, then the pre-exposure scores were used as a covariate in more complex models (e.g., [29]). Lastly, some studies compared the groups on a change score (post minus pre, e.g., [67] or nature minus built, e.g., [75] to further investigate associations between individual characteristics and positive response to nature.

#### 3.3.5. Studies’ Findings

##### Affective and Cognitive Outcomes

Of all 60 studies, approximately 88% (*n* = 53) found significant improvements in affect and restorative feelings following exposure to natural environments compared to built environments. Sixteen experiments also found worsened affective outcomes following the exposure to the built environment compared to baseline. Improved affective outcomes were observed after a short exposure to natural environments (as short as 15 min) either when walking in or viewing the environment. Even a short washout period (e.g., both sessions conducted on the same day) in within-subject studies appeared to be sufficient. In the 19 experiments investigating cognitive outcomes, findings were mixed, with only 10 (52.6%) yielding significant improvement in cognitive outcomes in the natural environment compared to the built environment. This may be related to the variation in the duration of the exposure, the washout period, and the tasks administered.

The studies that investigated cognitive outcomes used more varied durations of exposure and washout. Five experiments with longer durations (i.e., 50–55 min) in nature yielded significantly improved cognitive performance than in urban settings. Four studies with medium durations (25–40 min) yielded mixed results. Ten studies used shorter durations (5–20 min), of which most failed to yield a cognitive improvement. The two time-related tasks did yield significant differences between the environments, even with medium (time awareness [38]) and short (delay discounting [82]) durations. In Gidlow et al. [41], the exposure was of medium duration (30 min), and the cognitive performance was assessed three times, pre and post exposure, and 30 min after the end of the exposure. The researchers found that the cognitive improvement lasted 30 min after the exposure in the natural environment ended, whereas following the urban setting, at the follow-up measurement there was a decline in performance. This was the only study that performed a third measurement at follow-up, after a longer duration. Thus, it is possible that there is a minimum duration of exposure needed for improvement in cognitive performance. It is also possible that shorter exposures in nature can be sufficient; however, the improvement in performance manifests after some delay. Regarding washout periods between sessions in the within-subject studies, the two studies that utilized a short washout period (where the two sessions were conducted on the same day) did not result in significant cognitive differences between the conditions. In contrast, most of the studies that utilized a longer washout period (i.e., one week) appeared to result in significant improvements in the nature (vs. built) condition.

##### Other Outcomes

Only a few studies investigated other psychological outcomes. In general, natural environments, compared to built, appeared to affect time awareness [38], reduce delay discounting [82], decrease rumination [36], increase creativity (although not significantly) [81], increase connectedness to nature and the ability to reflect [54], and increase mindfulness [27]. For qualitative outcomes, Shrestha et al. [69] organized the responses into three themes, which were also supported by the other two qualitative studies, and generally aligned with the literature. The nature experience was described as enhancing positive affect (e.g., happiness, appreciation, gratitude, relaxation), enhancing restorative feelings (e.g., feeling physically and mentally energized, decreased discomfort), and enabling reflection, self-awareness, and present-moment orientation state (e.g., mindfulness, awe, clear thinking).

##### Underlying Mechanisms

Beyond the direct effect of the environment on the affective or cognitive outcomes, several studies also investigated involved factors or mechanisms—(a) individual characteristics, (b) the situational experience in the environment (e.g., level of the participant’s immersion, environmental conditions), and (c) associations among outcomes or other experimental factors.

- Individual Characteristics

Several studies investigated personal characteristics as potential moderators. Geniole et al. [40] found that men lower in nature connectedness benefited only from nature walks, whereas men higher in nature connectedness benefited from walking in both natural and built environments. The authors suggested that people who are high (versus low) in nature connectedness notice natural elements even when sparse, and thus can benefit even from low levels of vegetation that exist in urban neighborhoods. Ojala et al. [56] looked into urban–nature orientation and noise sensitivity and found that people who are nature-oriented and more sensitive to noise were not energized/refreshed in the historical downtown (like those who were more urban-oriented and less sensitive to noise) but were mostly affected in the more forested park (even compared to an urban park). Song et al. [75] found that higher trait anxiety scores were associated with a greater decrease in depression/dejection scores following the nature walk. Van der Wal et al. [82] found no association between growing up or current residence in an urban or rural environment and the effect of nature on delay discounting or change in mood.

- Situational Experience

Studies investigated whether and how the experience in the environment could explain the effect. Ballew et al. [28] found that the level of participant’s absorption in the environment mediated positive emotions and awe. Stigsdotter et al. [78] found that greater perceived restoration was related with improved mood. Similarly, Beil and Hanes [29] suggested that the perceived restoration, but not the environmental self-identity, predicted reduced levels of stress. Regarding environmental conditions, Park et al. [62,63] found that thermal comfort related to environmental conditions (i.e., illumination, humidity, atmospheric pressure) was related to improved mood.

- Associations Between Outcomes/Other Factors

Some studies analyzed associations between affective and cognitive outcomes, individual characteristics, or experimental factors. Berman et al. [30,31] found no correlation between mood and cognitive improvements, suggesting separate mechanisms. Similarly, Mayer et al. [54] found that nature connectedness, but not attention, mediated the effect of nature on the affective state, also suggesting the effects of nature on mood and attention occur via two separate mechanisms. In Stenfors et al.’s analysis with several within-subject studies [21], an association between affect and cognitive performance (specifically directed attention) was not found. However, Stenfors et al. [21] found an order effect in which the results depended on the order of the sites the participants experienced. Results showed that in the first session there were improvements in performance from pre to post walk in both environments. However, in the second session, the cognitive performance continued to improve only for participants who walked in the natural environment, whereas it declined for those who walked in the urban environment. The authors suggested that the natural environment was restorative, whereas the urban environment revealed a fatigue that was detrimental to cognitive performance.

## 4. Discussion

### 4.1. Summary of Evidence

The purpose of the current review was to synthesize past research of experiments conducted outdoors on the effect of natural vs. built environments on psychological outcomes to inform future studies on methodological practices. Several limitations, gaps, and innovative approaches were noted.

#### 4.1.1. Studies’ Samples

Most of the samples were not diverse or representative of the general population as most of the studies were conducted with young healthy male university students. Although more recent studies appear to address the gender imbalance that was more prevalent in older studies, additional research with mixed-gender, older, and clinical samples is needed (e.g., people with anxiety, post-traumatic stress disorder, chronic or acute pain, and substance use disorders).

#### 4.1.2. Experimental Sites and Environmental Conditions

Most studies used dichotomously opposite environments (e.g., beautiful green nature versus busy commercial urban site). Some recent studies focused on specific types of natural sites such as state parks, urban parks, blue spaces (with a water body), or nature sites during different seasons. For the purposes of this review, which reflects the extant literature in the reviewed area, all natural environments are treated as one category. Together, nature appears to be beneficial regardless of the type of natural elements in the environment (i.e., green, blue, white snow) or level of the vegetation (although higher levels of vegetation might be more beneficial for some individuals). The urban-built sites were more varied and less studied, warranting additional research on different types of urban environments, especially given the aesthetic diversity both across and within cities. Few studies examined urban areas that may be considered more restorative (e.g., historic downtown, residential neighborhood), or urban sites that have more or less natural elements. Understanding the impact of urban settings that have some level of vegetation or other urban elements on psychological outcomes can inform city planning and health education, as well as facilitate interpretation of research results. It is possible that beautiful urban areas, with unique architecture, or urban spaces that carry personal meaning, can also be restorative and beneficial for some people. Thus, focusing on the individual’s perceptions of the experimental site may be informative. Beil and Hanes [29] recommended including a measure of subjective restorative experience, as other environments or experiences may be restorative (e.g., art, architecture), and the effect of the environment may depend on the individual’s experience and preferences.

As the literature accumulates, future research should focus on understanding whether and how different types of vegetation and green/blue/other spaces affect different psychological outcomes. Future research explicitly designed to test the differences between variations in natural and built environments (including natural elements in built environments) will be needed to tailor environments to maximize specific psychological benefits for specific individuals. We also recognize the difficulty in conducting such experiments, requiring more resources, larger sample sizes, and access to multiple types of environments. Future research would benefit from developing standardized measures to describe the different natural and human-made elements in the experimental sites, as the exact dose of vegetation and greenness that yields beneficial effects is still unknown. Moreover, built environments may include more or less natural materials, which could affect psychological states and should be explored in future research.

Careful attention should be given to measuring and reporting environmental conditions beyond general weather conditions (e.g., “sunny”), as these should be standard assessments in environmental research. Standard reporting will enable comparison of findings across study locations, especially from very hot and humid areas. Qualitative descriptions are also informative and may be considered (e.g., traffic noise, chatter, music) in addition to quantitative measurements (e.g., sound level).

#### 4.1.3. Experimental Considerations

Several experimental procedures that warrant researchers’ attention were noted.

##### Baseline Measurements

Pre- and post-exposure measurements can provide more information than post-only measurements, in both within- and between-subject designs, and help interpretation. Pre/post measurements can inform of any detrimental effect of the built environment, rather than assuming a beneficial effect of the natural environment. Moreover, not all urban sites are equal, as some urban areas have some green, historical, or artistic elements and may be restorative to some individuals. However, pre- and post-measurements may not always be optimal. For example, regarding behavioral tasks (e.g., decision-making tasks), repeating a choice task in a short period might confound an environmental effect. A baseline measurement more distant in time from the experiment’s environmental exposure may be superior.

##### Masking

Most of the studies did not mask their participants. Although masking might be more difficult in within-subject studies, more attention should be given to the issue to minimize demand bias, as expectancies may enhance the effects.

##### Sense of Safety

In many of the studies, participants were instructed not to speak with others; however, they were often walking or viewing the environment in small groups (e.g., [32]) or led by a guide who set the pace of the walk (e.g., [39]). As such, although it was not directly discussed, these procedures may have provided a sense of safety, even if the nature experience itself was in solitude. Group context aligns with past research that feeling safe in a natural environment that is tended and open is important and leads to better psychological outcomes (e.g., [107,108]). Thus, future studies are encouraged to report on the instructions given to participants, the sense of safety, and the social context that are integral to the experience.

#### 4.1.4. Studied Outcomes

Only two studies included tasks that did not assess affective or cognitive performance per se (i.e., emotion, memory, attention). These studies utilized time-related tasks and provided initial evidence for the effect of nature on time perception and temporal discounting. The scarcity of studies utilizing behavioral tasks restricts specific methodological recommendations (e.g., duration of environmental exposure). More experimental outdoor research is needed on health- and behavior-related outcomes known to be affected by nature, such as pain, empathy, social interaction, and decision-making processes.

#### 4.1.5. Individual Differences as Moderators

Several studies reported that only a particular percentage of their participants responded positively to nature; however, most of the studies did not examine why different people respond differently. The studies that examined individual differences provided initial evidence that nature connectedness, urban orientedness, noise sensitivity, or trait anxiety were related to how one responds to nature. The question remains whether nature is beneficial only for those who enjoy/like it. Few studies included measures of the participant’s typical exposure to natural spaces. Pratiwi et al. [65,66] used such data to ensure similarity between groups, but not how they are related to nature’s effect. Typical exposure may be reflective of how the individual is connected, not connected, or even dislikes natural environments. Some people dislike nature due to a fear of animals/bugs. Whether these individuals may benefit from certain natural spaces is not a given, and this should be studied carefully in future research. Van der Wal et al. [82] included a measure of childhood or current greenness of a residential environment and found no association with temporal discounting. Understanding the personal characteristics involved is required to inform the development of individualized, evidence-based practice of nature prescription.

#### 4.1.6. Order Effect and Repeated Exposures

Stenfors et al. [21] revealed an order effect, in which nature was more beneficial for cognitive performance in the second session. In the first session, in both natural or built environments, participants’ performance improved from pre- to post-exposure due to training effects, as suggested by the authors. However, in the second session, following the nature walk, the cognitive performance continued to improve, while the performance following the urban walk worsened. The authors suggested that the built environment revealed mental fatigue that was detrimental to cognitive performance, whereas the natural environment was restorative and facilitated additional improvements. Future research should be powered to detect changes, considering any order effect that might exist (e.g., analyses requiring smaller sub-samples), and only certain percentages of participants positively respond to nature.

### 4.2. Limitations

The present scoping review has two main limitations. First, the article screening was conducted by a single researcher. To increase the rigor of the review, the screening stage was repeated and completed twice, to ensure the inclusion of all relevant studies. Second, the digital search failed to detect ten articles that were manually added to the current review. To understand why these articles failed to appear in the digital search, the ten articles were scanned. This process suggested valid reasons as the ten articles included terms that were set to be excluded such as ‘laboratory’, ‘video’, or ‘children’, or the psychological outcomes were not mentioned in the title and abstract. Still, we were able to locate 60 articles, which gave us confidence in the extensiveness of this scoping review.

## 5. Conclusions

The current review aimed to form updated methodological recommendations to advance outdoor experiments, which offer a more holistic experience of nature than laboratory experiments. With a call for more research with understudied and new outcomes, this line of research has important implications for health education, city planning, nature prescription, and treatment programs (e.g., substance use treatment). To better understand the optimal “dose-response” and advance the utilization of nature in health-promoting practice, specific methodological recommendations for researchers and future directions are as follows:

1. Recruit larger and more diverse samples to enable generalization, investigation of unique subgroups of participants and proportions of positive responders, and order effects in within-subject studies.
2. Mask participants when possible. Describe the study with general terms like “environments” or “outdoors” rather than using the word “nature.”
3. Include pre- and post-exposure measurements when possible. A baseline measurement (either indoors or at the experimental site) can help determine the effect of each environment independently and in comparison to one another.
4. Include environmental measures (e.g., temperature, humidity, noise) to control for in the analysis, compare the conditions between the environments, or examine associations with outcomes. Assess and report visual, sound, and smell characteristics and evaluate congruence between environments (e.g., an urban plaza might be very green but noise-polluted with heavy traffic). Related to the sense of touch, beyond the environmental conditions (e.g., wind, temperature, humidity), report elements related to the sensation of walking (e.g., asphalt, concrete pavement, forest trails) or sitting (e.g., ground, chair).
5. For improvements in affective outcomes, shorter durations of viewing or walking in the environment may be sufficient (e.g., 15 min), as well as shorter washout periods between sessions in within-subject designs. For cognitive performance, it appears that longer durations are required (40–60 min) with longer washout periods between sessions (e.g., one week apart). For behavioral tasks and other measures, the duration of exposure and washout periods are unknown as more research is needed.
6. To understand individual differences, include baseline nature-related measures (e.g., nature connectedness, typical time spent in nature, residential greenness), as well as measures of the situational perceived experience (e.g., restorative experience, perceived beauty, immersion), or other potential mediators, and test for associations with outcomes.
7. For the experimental environments, ensure the natural environment is open, safe, and rich with green or blue elements. Ensure the urban environment is low on green elements yet offers a safe and potentially restorative urban experience (e.g., beautiful architecture, open space). Moving away from the dichotomous green–gray environments and examining urban areas with different levels of vegetation will offer a better understanding of the therapeutic potential of urban environments.
8. Future research should investigate understudied clinical populations (e.g., people with chronic pain conditions, substance use disorders) and explore other outcomes utilizing behavioral tasks and other measures, for example, social outcomes, pain, and addictive behaviors.

## Figures and Tables

**Table 1:** Study characteristics: location of study, sample, design, activity, outcome measures, environmental conditions, and results of the included studies.

	Study First Author, Year Location	Sample *N* (*n* Female), Mean Age, Description	Design Between/Within (Washout Period), Measurements	Activity Type, Duration of Interaction with Environment	Environmental Conditions	Outcome Measures		Results	
					Measures/Analysis	Self-Reported Scales	Tasks	Did Nature (vs. Built) Improve Outcomes?	Did Built Worsen Outcomes (Pre vs. Post)?
#1	Abdul Aziz et al., 2021 [26] Malaysia	90 (45), 22.9 y, healthy university students	Between, pre-post	Walk, 20 min	Weather, temperature, humidity, wind/ descriptive	POMS, PANAS, ROS	-	Affective: Yes Restorative: Yes	Yes (n.s.)
#2	Ameli et al., 2021 [27] U.S.	12 (9), 35 y, military personnel	Within (within 1 week), pre-post	Walk, 20 min	None	Qualitative, Distress VAS, MAAS	-	Affective: Yes	No
#3	Ballew & Omoto, 2018 [28] U.S.	100 (55), 19.3 y, healthy university students	Between, post	View, 15 min	None	Absorption, Awe, Positive Emotions	-	Affective: Yes Restorative: Yes	N/A
#4	Beil & Hanes, 2013 [29] U.S.	15 (7), 42.3 y, community members	Within (non-consecutive days), pre-post	View, 20 min	None	Subjective Stress VAS, PRS	-	Affective: Yes	No
#5	Berman et al., 2008 (Exp. 1) [30] U.S.	38 (23), 22.6 y, healthy university students	Within (2 weeks), pre-post	Walk, 50–55 min	Season/ covariate	PANAS	BDS	Affective: Yes Cognitive: Yes	No
#6	Berman et al., 2012 [31] U.S.	20 (12), 26.0 y, adults with depression	Within (1 week), pre-post	Walk, 50–55 min	None	PANAS	BDS	Affective: Yes Cognitive: Yes	No (affect improved in both) (cog. Yes n.s.)
#7	Bielinis et al., 2018 [32] Poland	62 (26), 21.5 y, healthy university students	Between, pre-post	View, 15 min	Weather, temperature, humidity, wind, noise/ descriptive	POMS, SVS, PANAS, ROS	-	Affective: Yes Restorative: Yes	Yes
#8	Bielinis et al., 2018 [33] Poland	54 (24), 21.4 y, healthy university students	Between–environment Within–seasons pre-post	View, 15 min	Weather, temperature, humidity, wind, noise/ descriptive	POMS, SVS, PANAS, ROS	-	Affective: Yes Restorative: Yes (more in winter)	Yes (more in spring)
#9	Bielinis et al., 2019 [34] Poland	32 (32), 21.0 y, healthy university students	Between, pre-post	View, 15 min	Weather, temperature, humidity, wind, noise/ descriptive	POMS, SVS, PANAS, ROS	-	Affective: Yes Restorative: Yes	Yes
#10	Bielinis et al., 2021 [35] Finland	22 (11), 22.5 y, healthy university students	Within (same day), pre-post	View, 15 min	Weather, temperature, humidity, wind, noise/ descriptive	POMS, SVS, PANAS, ROS	-	Affective: Yes	Yes
#11	Bratman et al., 2015 [36] U.S.	60 (33), 22.9 y, community members and students	Between, pre-post	Walk, 50 min	None	PANAS, STAI, RRQ	BDS, OSPAN, ANT, Change detection task	Affective: Yes Cognitive: Yes (mixed)	Yes (affect)
#12	Chen et al., 2016 [37] China	32 (16), 20.6 y, healthy university students	Between, pre-post	View, 20 min	Weather, temperature, humidity, wind, noise/ descriptive	POMS, PRS	Necker Cube Pattern Control Test	Affective: Yes Cognitive: No	Not reported
#13	Ehret et al., 2020 [38] Germany	48 (35), 26.4 y, healthy university students and community members	Between–environment. Within–tasks, (3 separate days), pre-post (mood), post (tasks)	Passive (sit), 25–35 min	Weather, temperature/ descriptive	SAMS, MDBF, Qualitative	Time awareness VAS, PoTJ VAS, Time Estimation	Time awareness: Yes Affective: Yes	Yes
#14	Elsadek et al., 2019 [39] China	364 (164), 23.0 y, healthy university students and visitors	Within (5 min), pre-post	Walk, 15 min	Weather, temperature, humidity, wind, solar radiation, noise/ descriptive	POMS, STAI, ROS, SVS	-	Affective: Yes Restorative: Yes	Yes
#15	Geniole et al., 2016 [40] Canada	31 (0), 24.6 y, healthy university students	Within (within 1 week), pre-post	Walk, 25 min	Weather, temperature/ comparison	Affect Grid	Stroop	Affective: Yes Cognitive: No	No
#16	Gidlow et al., 2016 [41] UK	38 (15), 40.9 y, community members	Within (separate days within two weeks), pre-post, follow up 60 min	Walk, 30 min	Noise/ Descriptive, comparable	BRUMS, ROS	BDS	Affective: No (all improved), Restorative: Yes Cognitive: Yes (in follow up)	No
#17	Hartig et al., 1991 (Exp. 2) [42] U.S.	34 (17), 20.0 y, healthy university students	Between, pre-post	Walk, 40 min	None	ZIPERS, OHS, PRS	Proofreading task	Affective: Yes Restorative: Yes Cognitive: Yes	Not performed
#18	Hartig et al., 1999 (Exp. 3) [43] U.S.	101 (62), 20.6 y, healthy university students	Between, post	View, 15 min	Weather/ comparison	PANAS, ZIPERS	Mood-congruence memory tests	Affective: Yes (partly) Cognitive: No	N/A
#19	Hassan et al., 2018 [44] China	60 (30), 19.6 y, healthy university students	Within (2 consecutive days), pre-post	Walk, 15 min	Weather, temperature, humidity/ descriptive	SDM, STAI	-	Affective: Yes	Not performed
#20	Igarashi et al., 2015 [45] Japan	17 (17), 46.1 y, urban community members	Within (no washout), post	View, 10 min	Weather, temperature, humidity, illumination/ descriptive	SDM, POMS	-	Affective: Yes	N/A
#21	Joung et al., 2015 [46] Korea	7 (not reported), 22.0 y, healthy university students	Within (not reported, fixed order nature → urban), post	View, 15 min	None	SDM, POMS	-	Affective: Yes	N/A
#22	Joung et al., 2020 [47] Korea	24 (9), 20.8 y, healthy university urban students	Within (4 days, fixed order nature → urban), post	Walk, 15 min	Weather/ descriptive	SDM, POMS, STAI	-	Affective: Yes	N/A
#23	Kabisch et al., 2021 [48] Germany	33 (21), 63.5 y, older adults	Within (separate days over 9 days, fixed order: old park → street → new park), pre-post	View and Walk, 15 + 30 min	Weather/ descriptive	POMS, ROS	-	Affective: Yes (n.s.) Restorative: Yes	Yes
#24	Lee et al., 2009 [49] Japan	12 (0), 21.3 y, healthy university students	Within (2 consecutive days), pre-post	View, 15 min	Temperature, humidity/ descriptive	SDM, Stress and Arousal Scale	-	Affective: Yes	Not performed
#25	Lee et al., 2011 [50] Korea	20 (0), 24.0 y, healthy university students	Within (2 consecutive days), post	View, 15 min	None	SDM, POMS, STAI, SCL-90-R	-	Affective: Yes	N/A
#26	Lee et al., 2011 [51] Japan	12 (0), 21.2 y, healthy university students	Within (2 consecutive days), pre-post	View, 15 min	Weather/ descriptive	SDM, POMS	-	Affective: Yes	Yes
#27	Lee et al., 2014 [52] Japan	48 (0), 21.1 y, healthy university students	Within (2 consecutive days), pre-post	Walk, 12–15 min	Weather/ descriptive	SDM, POMS, STAI, Stress and Arousal Scale	-	Affective: Yes	Not performed
#28	Lee et al., 2015 [53] Japan	12 (0), 22.3 y, healthy university students	Within (2 consecutive days), pre-post	View, 15 min	Weather/ descriptive	SDM, POMS, Stress and Arousal Scale	-	Affective: Yes	Not performed
#29	Mayer et al., 2009 (Study 1) [54] U.S.	76 (51), not reported, healthy university students	Between, post	Walk and View, 10 + 5 min	Weather/ descriptive, comparable	PANAS, SSAS, CNS, Ability to reflect single-item	Search Task	Affective: Yes Cognitive: Yes Connectedness to nature: Yes Ability to reflect: Yes Self awareness: No	N/A
#30	Nicolosi et al., 2021 [55] U.S.	63 (31), 20.4 y, healthy university students	Within, (same day), post	Walk, 10–15 min	Weather, temperature, wind, noise/ descriptive	SRRS	Quiz on reading	Affective: Yes Restorative: Yes Cognitive: No	N/A
#31	Ojala et al., 2019 [56] Finland	83 (83), 48.3 y, community members	Within (at least one week), pre-post	View and Walk, 15 + 30 min	Weather, temperature/ descriptive Noise/ comparison	ROS, SVS, Nature-urban oriented, Noise sensitivity	-	Affective: Yes	Yes
#32	Olszewska-Guizzo et al., 2020 [57] Singapore	22 (13), 32.9 y, community members	Within (average 11 days), pre-post	View, 30–45 min (6 min. physiological)	Temperature, humidity, brightness, noise/ covariate	POMS	-	Affective: No	Yes (trend)
#33	Olszewska-Guizzo et al., 2022 [58] Singapore	92 (52), 36.6 y, depressed and healthy adults	Within (average 9 days), pre-post	View, 30–45 min (6 min. physiological)	Temperature, humidity, brightness, noise/ covariate	POMS	-	Affective: Yes	Yes
#34	Park et al., 2007 [59] Japan	12 (0), 22.8 y, healthy university students	Within (2 consecutive days), pre-post	Walk (morning) and View (afternoon), 20 + 20 min	None	SDM	-	Affective: Yes	Not performed
#35	Park et al., 2008 [60] Japan	12 (0), 21.3 y, healthy university students	Within (2 consecutive days), pre-post	View, 15 min	None	SDM	-	Affective: Yes	Not performed
#36	Park et al., 2009 [61] Japan	12 (0), 21.8 y, healthy university students	Within (2 consecutive days), pre-post	Walk (morning) and View (afternoon), 15 + 15 min	None	SDM	-	Affective: Yes	Not performed
#37	Park et al., 2010 [62] Japan	280 (0), 21.7 y, healthy university students	Within (2 consecutive days), pre-post	Walk (morning) and View (afternoon), 15 + 15 min	Temperature, humidity, radiant heat, wind, atmospheric pressure, illumination/ Association with outcomes	POMS	-	Affective: Yes	Not performed
#38	Park et al., 2011 [63] Japan	168 (0), 20.4 y, healthy university students	Within (2 consecutive days), pre-post	View (morning) and Walk (afternoon), 15 + 15 min	Temperature, humidity, radiant heat, wind, atmospheric pressure/ Comparison, association with outcomes	SDM, POMS	-	Affective: Yes	Yes
#39	Perkins et al., 2011 [64] U.S.	26 (19), range 19–24 y, healthy university students	Between, pre-post	Walk, 20 min	None	POMS	BDS, Digit span forward, logical memory	Affective: No (all conditions improved) Cognitive: No	No
#40	Pratiwi et al., 2019 [65] Japan	24 (13), Spring: 66.4 y, Summer: 65.8 y, older adults	Within (same day morning, afternoon), pre-post	View, 11–15 min	Temperature, humidity/ descriptive	POMS, STAI	-	Affective: Yes	Not performed
#41	Pratiwi et al., 2020 [66] Japan	36 (21), winter: 70.2 y, spring: 66.4 y, summer: 66.8 y, middle aged and older adults	Within, (same day morning, afternoon), pre-post	Walk, 11–15 min	Temperature, humidity/ descriptive	POMS, STAI	-	Affective: Yes	Not performed
#42	Rider & Bodner 2016 (Exp. 1) [25] Canada	24 (20), 22.0 y, healthy university students	Within, (same day), post	Walk, 10 min	None	Mood (single item)	Memory: free recall/recognition	Affective: No Cognitive: No	N/A
	Rider & Bodner 2016 (Exp. 2) [25] Canada	24 (19), 22.0 y, healthy university students	Within (same day), post	Walk, 11 min	None	Mood (single item)	Memory: free recall/recognition	Affective: No Cognitive: No	N/A
#43	Roe et al., 2020 [67] U.S.	11 (5), 64.8 y, older adults	Within, (1 day between), pre-post	Walk, 15–20 min	Weather, noise, air pollution/ Comparison, association with outcomes	MACL	Deary-Liewald Cognitive reaction time, Memory recall of route	Affective: Yes Cognitive: Yes (n.s.)	Yes (n.s.)
#44	Shin et al., 2011 [68] Korea	60 (25), 23.3 y, healthy university students	Within (1 week), pre-post	Walk, 50–55 min	None	POMS	Trail Making Test Part B	Affective: Yes Cognitive: Yes	Yes (affect)
#45	Shrestha et al., 2021 [69] Ireland	13 (10), 24.9 y, healthy university students	Between, post	Walk, 20–25 min	Weather, noise type/ descriptive	Qualitative, PRS-11	-	Affective: Yes Restorative: Yes	Yes (qualitative)
#46	Song et al., 2013 [70] Japan	13 (0), 22.5 y, healthy university students	Within (20 min), post	Walk, 15 min	Weather, temperature, humidity, illumination/ descriptive	POMS, STAI, SDM	-	Affective: Yes	N/A
#47	Song et al., 2014 [71] Japan	17 (0), 21.2 y, healthy university students	Within (20 min), post	Walk, 15 min	Weather, temperature, humidity/ descriptive	POMS, STAI, SDM	-	Affective: Yes	N/A
#48	Song et al., 2015 [72] Japan	20 (0), 58.0 y, middle aged hypertensive	Within (2 consecutive days), post	Walk, 17 min (morning)	Weather, temperature, humidity/ descriptive	POMS, SDM	-	Affective: Yes	N/A
#49	Song et al., 2015 [73] Japan	23 (0), 22.3 y, healthy university students	Within (20 min), post	Walk, 15 min	Weather, temperature, humidity, illumination/ descriptive	POMS, STAI, SDM	-	Affective: Yes	N/A
#50	Song et al., 2017 [74] Japan	20 (0), 58.0 y, middle aged hypertensive	Within (2 consecutive days), post	View, 10 min (afternoon)	Weather, temperature, humidity/ descriptive	SDM	-	Affective: Yes	N/A
#51	Song et al., 2018 [75] Japan	585 (0), 21.7 y, healthy university students	Within (2 consecutive days), post	Walk, 15 min	None	POMS, STAI	-	Affective: Yes	N/A
#52	Song et al., 2019 [76] Japan	60 (60), 21.0 y, healthy university students	Within (2 consecutive days), post	Walk, 15 min	Weather, temperature, humidity, illumination/ descriptive	POMS, STAI, SDM	-	Affective: Yes	N/A
#53	Song et al., 2019 [77] Japan	65 (65), 21.0 y, healthy university students	Within (2 consecutive days), post	View, 15 min	Weather, temperature, humidity, illumination/ descriptive	POMS, STAI, SDM	-	Affective: Yes	N/A
#54	Stenfors et al., 2019 (unpublished data 2016) [21] U.S.	49 (31), 19.6 y, healthy university students	Within (1 week), pre-post	Walk, 20 min	None	PANAS	BDS	Affective: not reported Cognitive: No	Not reported
	Stenfors et al., 2019 (unpublished data 2011) [21] U.S.	21 (11), 23.6 y, healthy university students	Within (1 week), pre-post	Walk, 50–55 min	None	PANAS	BDS	Affective: not reported Cognitive: Yes (nature first)	Not reported
#55	Stigsdotter et al., 2017 [78] Denmark	51 (51), range 20–36 y, healthy university students	Within (separate days within two weeks), pre-post	Walk and Rest, 15 + 5 min	None	POMS, PRS	-	Affective: Yes Restorative: Yes	No
#56	Takayama et al., 2014 [79] Japan	45 (0), 20.8-21.4 y, healthy university students	Within (2 consecutive days), pre-post	Walk (morning) and View (afternoon), 15 + 15 min	Weather, temperature, humidity/ descriptive	POMS, SVS, PANAS, ROS	-	Affective: Yes Restorative: Yes	Yes
#57	Tsunetsugu et al., 2007 [80] Japan	12 (0), 22.0 y, healthy university students	Within (2 consecutive days), pre-post	Walk (morning) and View (afternoon), 15 + 15 min	Weather, temperature, humidity/ descriptive	SDM	-	Affective: Yes	Yes
#58	Tyrväinen et al., 2014 [81] Finland	77 (71), 47.6 y, community members	Within (at least one week), pre-post	View and Walk, 15 + 30 min	Weather, temperature/ descriptive	SVS, PANAS, ROS, PRS, Creativity	-	Affective: Yes Restorative: Yes Creativity: No	Yes
#59	van der Wal et al., 2013 [82] (Exp. 3) Netherlands	43 (26), 31.8 y, community members	Between, post	Walk, 5 min	None	Future Valuation, Self-Control, Mood	Delay Discounting	Affective: Yes Delay Discounting: Yes	N/A
#60	Watkins-Martin et al., 2022 [83] Canada	37 (25), 49.3 y, adults with depression	Between, pre-post	Walk, 60 min	Weather/ Descriptive, comparable	PANAS	-	Affective: Yes	Yes

*Note*. Environmental conditions: “weather” = a general description (e.g., sunny, cloudy); “descriptive” = descriptive report; “comparable” = authors report the conditions in the environments as comparable; “comparison” = environments were statistically tested for difference; “association with outcomes” = correlation testing between conditions and outcomes; “covariate”—conditions tested as confounding factor. Affect Grid [84]; ANT: Attention Network Test; BDS: Backward Digit Span; BRUMS: Brunel Mood Scale [85]; CNS: The Connectedness to Nature Scale [86]; MAAS: Mindful Attention Awareness Scale [87]; MACL: Mood Adjective Check List [88]; MDBF: German Multidimensional Mood State Questionnaire [89]; N/A: not-applicable; n.s.: non-significant; OHS: Overall Happiness Scale [90]; OSPAN: Operation Span Task; PANAS: The Positive and Negative Affect Schedule [91]; POMS: The Profile of Mood States [92]; PoTJ: Passage of Time Judgements; PRS: Perceived Restorativeness Scale [93]; ROS: The Restorative Outcome Scale [94]; RRQ: Rumination-Reflection Questionnaire [95]; SAMS Self-Assessment Manikin Scale [96]; SCL-90-R: Symptom checklist-90-Revision [97]; SDM: Semantic Differential Method [98]; SRRS: Short-version Revised Restoration Scale [99]; SSAS: The Situational Self-Awareness Scale [100]; STAI: State Trait Anxiety Inventory [101]; Stress and Arousal Scale [102]; SVS: The Subjective Vitality Scale [103]; VAS: Visual Analogue Scale; ZIPERS: The Zuckerman Inventory of Personal Reactions [104].

## Data Availability

The original contributions presented in this study are included in the article. Further inquiries can be directed to the corresponding authors.

## Appendix A

The final search terms used in Web of Science were as follows:

Topic:

(Natur* OR green OR park OR parks OR forest* OR “shinrin-yoku”) AND (Urban* OR city OR cities OR traffic OR building) AND (outdoor* OR environment* OR setting* OR reserve* OR surrounding* OR feature* OR landscape* OR area OR areas OR space*) AND (Interact* OR walk* OR view* OR visit* OR watch* OR expos* OR contact) AND (Mood OR feeling* OR anxiety OR stress* OR cortisol OR affect* OR emotion* OR cognitive OR attention) AND (experiment* OR control* OR randomised OR randomized).

NOT (All fields):

exercis* OR fitness OR jog OR jogs OR jogging OR run OR runs OR running OR image OR images OR picture* OR virtual OR child OR adolescent OR children OR teenager.
